# A Longitudinal Perspective Case Study of Delusional Parasitosis in a Geriatric Psychiatry Unit

**DOI:** 10.7759/cureus.39434

**Published:** 2023-05-24

**Authors:** Anisa Suparmanian, Nathan J Cardona

**Affiliations:** 1 Psychiatry, The Wright Center for Graduate Medical Education, Scranton, USA; 2 Research, The Wright Center for Graduate Medical Education, Scranton, USA

**Keywords:** geriatric psychiatry, convulsive therapy, psychopharmacology, schizophrenia, delusional parasitosis

## Abstract

Delusional parasitosis is not a common presentation in hospital-based geriatric units. Our aim was to review the presentation of a sudden onset of parasitosis in an older patient who had no prior psychiatric history, and its management. This case report describes an 82-year-old man who presented with delusions of parasitosis for the past three years of his life. The report includes a longitudinal description of the patient's symptoms, signs, and manifestations during his stay at an inpatient senior mental health service center, emergency department (ED) visits, and medical floor stay along with medication management of his psychiatric symptoms with concomitant medical issues. Presentation of delusional parasitosis poses a special challenge not only psychiatrically but also medically and dermatologically. The unique difficulty of finding appropriate antipsychotics for not only the symptoms of parasitosis but also the compulsive behavior that follows a deeply ingrained belief of being infested must be carefully managed, especially in the elderly. Somatic delusions that resemble a plausible but inaccurate reality of infestations could delay treatment as well.

## Introduction

Delusional parasitosis is a rare presentation, yet remarkably remembered by practitioners from all fields who are involved in its related care. Data shows its incidence increases across the life span, especially after age 40, with an estimated incidence of 1.9 per 100 000 person-years [1-2]. Often, the patients’ reluctance to seek psychiatric care and late referrals result in delayed treatment. Here, we report the difficulty and challenges faced when treating patients at late-stage presentation, when physical deterioration from fixed false beliefs affect food intake and activities of daily living. The report shows the uniqueness of the presentation, and the pitfalls of treatment and management, along with a lengthy course of progression from minimal symptoms to full-blown parasitosis delusion.

## Case presentation

An 82-year-old gentleman presented to the emergency department in early 2021, with complaints of “bugs” crawling inside him and in his sputum. The patient reported that there were a variety of these insects, they originated from his stomach, and sometimes they went into his lungs, which caused him to cough. The patient was subsequently admitted to an inpatient psychiatric unit. 

The patient insisted on getting a room without carpets because he believed that the insects were crawling from out of them and into his body. He had multiple excoriation marks all over his body and displayed a flexed posture of his neck, which he could extend if asked. He believed that there were insects behind his neck and that they were biting him, but nothing was found upon examination. There were no focal neurological deficits. The patient described the bugs as “white” in color, mostly looking like silverfish and other varieties which he could not describe. The patient did have skin irritation caused by a wristband, but had misconstrued this with bug bites; possibly due to dubious vision. In the unit, the patient was continuously scratching himself, spitting, and excessively washing resulting in sores. When asked what would happen if he stopped himself from repeatedly scratching, he stated that he would become extremely anxious. 

The patient's history was given by a close family member, who reported that symptoms started subtly in early 2018 when the patient initially complained of bugs being around the home. During the emergence of these symptoms, the family member would agree to call exterminators, believing the patient's delusions to be true. The patient had seen many dermatologists for the itchiness and crawling sensation in his skin, which had not improved at the time of admission. The patient also had a long history of hospital admissions for multiple medical issues ranging from approximately monthly to once every eight months. His medical issues ranged from shingles to atrial fibrillation, episodes of syncope and falls, lower extremities edemas, and chest cellulitis. During most of his admissions, he had complained of bugs all over his body.

Finally, toward the end of 2020, his symptoms worsened to such a degree that he was admitted to the inpatient behavioral unit for the first time, for decompensation in his delusions. He was started on fluvoxamine 25 mg at night and aripiprazole 2 mg at night, which was increased to 10 mg at night. The patient's symptoms gradually improved, and he was discharged. Unfortunately, his non-compliance with medication at home brought him back to the inpatient unit five months later in early 2021, where we first encountered the patient.

Laboratory testing was done during his current admission, including vitamin B12, urine analysis, thyroid function, rapid plasma reagin, complete blood count, and metabolic panel. All were relatively normal. He was started on aripiprazole 10 mg at night again and fluvoxamine 50 mg twice a day; aripiprazole was gradually increased to 20 mg at night, but due to non-effectivity, it was discontinued. Olanzapine 10 mg at night was then started, then increased to 15 mg at night, but was stopped due to continued symptoms. 

Doctors then started the patient on thiothixene 5 mg at night and increased it to 15 mg, but it was discontinued due to extrapyramidal symptoms. When started on cariprazine 1.5 mg daily, and then 3 mg daily several days later, the patient became more irritable and so it was discontinued. His symptoms mostly waxed and waned. Alprazolam 0.5 mg was given three times daily, then increased to four times daily, which helped with his anxiety, and eventually was changed to lorazepam 0.5 mg twice a day. Fluvoxamine was increased to 50 mg in the morning and 100 mg at night. Occupational therapy was consulted to help him with his neck flexion, which showed an improvement several days later. The neurology team believed that he was exhibiting parkinsonism symptoms and Lewy body dementia because he showed minor psychomotor slowing and inattention, along with memory and processing impairment; this raised the possibility of dementia or the possibility of psychosis associated with Parkinson’s disease. Because of this, the patient was given pimavanserin 34 mg at night but it was stopped after two days due to increased agitation. 

No computed tomography (CT) scan was done during this latest admission, but evidence from past CT scans showed that he had prominence of the ventricles and sulci, which are both consistent with age-related atrophy. However, no further investigations or MRI of the brain were attempted. These scans suggested mild chronic microvascular ischemic changes, but no acute abnormalities in the temporal or occipital lobes. 

Finally, the patient underwent nine sessions of electroconvulsive therapy (ECT), which eventually helped him with his delusions to a certain degree, adherence to medications, and he started to eat a healthy amount again. However, his delusions were still present and it was decided that ECT should be terminated. The patient was then discharged home with fluvoxamine 50 mg in the morning and 150 mg at night, along with lorazepam 0.5 mg twice a day and 1 mg at night. He had a home health aide involved for a time, but eventually, he was moved into a nursing home. 

## Discussion

Delusional disorders fall under the category of schizophrenia spectrum and other psychotic disorders, in which delusional beliefs are fixed and not amenable to change, even in the presence of contrary evidence [3,4]. A criterion for delusional disorder is the presence of one (or more) delusions with a duration of one month or longer. Criteria for schizophrenia had never been met in this case, and the related hallucinations are connected to the theme of the delusions only. The function of the patient is not markedly impaired, except when it is directly due to the impact of the delusions itself [4].

Delusional parasitosis is a somatic type of delusional disorder, the theme of which involves the sensation of insect infestation on or in the skin, or an internal parasite [3,4]. Symptomatically, they can present with either delusions, chronic tactile or visual hallucinations, or a combination of the three [5]. Parasitosis can afflict patients for days to decades, with an average duration of three years [5]. Seventy-four percent of delusional parasitosis occurs in patients with a history of depression [5,6]. When patients misconstrue a real item with a false pathogen, the psychopathology could be classified as an illusion instead of a hallucination; this can worsen for patients with impaired vision [5,7].

The main modalities of treatment for delusional infestations (DI) are atypical antipsychotics like olanzapine, risperidone, or amisulpride, or a typical and relatively safe antipsychotic like haloperidol, sulpiride, or perphenazine [5].

The decision to start the patient on fluvoxamine 50 mg at night was empirically started for compulsive scratching. Aripiprazole was chosen due to its relatively mild side effects as a second-generation antipsychotic and, considering the patient's age, to reduce the risk of metabolic effects in lieu of multiple comorbid medical conditions. The patient's slow symptom recognition by practitioners may have been due to their assumption that real exposure to insects was an inherent part of engagement in his reported outdoor hobbies/work activity. Considering that even his close family member believed the bug infestations were real for a time, the patient’s symptoms seemed to have been masked quite consistently. The patient's reluctance to start himself on any neuroleptics was typical of studies showing that patients with delusional parasitosis commonly consult numerous physicians and even get help from pest control services rather than a psychiatrist [8,9].

There was a significant limitation in the course of the patient’s clinical care; it was difficult to identify whether an underlying neurocognitive decline was playing a role in his pathology. The neurologist that examined him suspected a possible neurocognitive decline; however, the patient failed to follow up in a neurocognitive clinic, and no medications were started for memory issues, nor did he get a full workup for it. Existing literature shows that DI is rare, and could present in dementia with Lewy bodies [10,11]. The patient experienced symptoms that had become so severe that he had started neglecting his activities of daily living, similar to that of patients with dementia. 

There were no reports of outpatient follow-up throughout the previous three years of symptom presentation, thus causing a knowledge gap as to how his presentation progressed prior to his first psychiatric admission in 2020. Before provider suspicions led to the first psychiatric admission, the focus on serious medical issues had shifted attention from his psychiatric complaints to life-threatening medical conditions that needed immediate attention. The clinical picture and progression of this case could have been more positive if, at an earlier stage, a referral to psychiatry was made or consult-liaison psychiatry services were involved. Integration of psychiatric care during the initial stages, along with a transition of care from consult-liaison psychiatric services to outpatient psychiatric follow-up, might have changed the outcome of this case. The involvement of neurology in patients with neurocognitive decline could help assist the team further in the complexities of the case presentation. Cognitive behavioral therapy (CBT) could also have proven to be useful, especially habit reversal therapy in psychodermatology.

Striato-thalamic-parietal circuitry may be relevant to the pathophysiology of DI [12,13]. However, MRI had not been obtained, which would have enabled a greater study of any changes in the patient’s brain. 

Many studies found that social isolation is a risk factor [14]. We believe this may have been a strong catalyst in our case, as the patient lived with only one family member and was vulnerable to social isolation during the coronavirus disease 2019 (COVID-19) pandemic lockdown.

## Conclusions

This case report highlights the propensity of healthcare professionals to delay referrals to the psychiatry team when a patient presents to the hospital with physical complaints. This is more often likely to occur if the somatic delusions resemble a plausible but inaccurate reality, thus delaying treatment. The presentation of delusional parasitosis poses a special challenge not only psychiatrically but also medically and dermatologically. The unique difficulty of finding appropriate antipsychotics for not only the symptoms of parasitosis but also the compulsive behavior that follows a deeply ingrained belief of being infested must be carefully managed, especially in the elderly.

The clinical picture and progression of this case could have been more positive if, at an earlier stage, a referral to psychiatry was made or consult-liaison psychiatry services were involved. Integration of psychiatric care during the initial stages, along with a transition of care from consult-liaison psychiatric services to outpatient psychiatric follow-up, might have changed the outcome of this case. The involvement of neurology in patients with neurocognitive decline could help assist the team further in the complexities of the case presentation.
